# The Senticaudata, a suborder of the Amphipoda – A commentary on d’Udekem d’Acoz and Verheye (2017)

**DOI:** 10.3897/zookeys.730.22126

**Published:** 2018-01-18

**Authors:** Alan A. Myers, Jim K. Lowry

**Affiliations:** 1 School of Biological, Earth and Environmental Sciences, University College Cork, Cork Enterprise Centre, Distillery Fields, North Mall, Cork, Ireland; 2 Australian Museum Research Institute, 6 College Street, Sydney, NSW 2010, Australia

**Keywords:** Amphipoda, classification, phylogeny, Senticaudata

## Abstract

A response is given to criticisms in a recent paper of the validity of the amphipod suborder Senticaudata. The tacitly assumed status of truth implied in some molecular higher phylogenies is called in to question.

## Introduction

The suborder Senticaudata was established by Lowry and Myers (2013) based on a number of character states, but primarily on the important synapomorphy of apical robust setae on the rami of uropods 1 and 2. This character occurs in nearly 100 families and more than 5,000 species which form the suborder. The Senticaudata is recognised by the WoRMS online database and is cited by most taxonomic papers reporting on taxa in the group. The paper has been cited in at least 115 publications (Google Scholar). It has recently been included in a new phylogenetic classification of the Amphipoda (Lowry and Myers 2017).

## Discussion

In a recent paper by d’Udekem d’Acoz and Verheye (2017), the validity of the amphipod suborder Senticaudata (Lowry and Myers 2013) is called into question. The argument put forward for questioning the status of the suborder, is that the main defining character state for this suborder proposed by Lowry and Myers (2013), the presence of apical robust setae on uropods 1 and 2, is also found to be present in a few taxa outside the Senticaudata. They comment:

*Indeed, senticaudate taxa do not form a clade, suggesting that the distal ornamentation of uropods would be subject to homoplasy.*

We absolutely agree that the said character state is homoplastic in some taxa and indeed we acknowledged this in our 2013 paper. However, the existence of homoplasies does not falsify a synapomorphy. The Senticaudata were not defined (as asserted by Verheye et al. (2016) by just “one alleged synapomorphy”. The study by Lowry and Myers (2017) was based on 115 characters in the cladistic analysis, although the presence of apical robust setae on uropods 1 and 2 was one of the most important. Senticaudates also lack complex callynophores and brush setae. The Senticaudata includes nearly 100 families and more than 5,000 species that universally display this character state. The Senticaudata erected by Lowry and Myers (2013) was not a revolutionary concept, the great majority of taxa included therein had traditionally been recognised as an interrelated group. It had simply not been given taxonomic ranking before. Only very few taxa, outside the Senticaudata, have apical robust setae on uropods 1 and 2. Lowry and Myers (2013) described the situation in the Haustoriidae, where the convergence was attributed to their burrowing life style. In the case of the Idunellinae, mentioned by d’Udekem d’Acoz and Verheye (2017), the environmental factors over evolutionary time, leading to the convergence in this subfamily, cannot be determined. However, in the other subfamily of the Liljeborgiidae, the Liljeborgiinae, there are no robust setae on the apices of uropods 1 and 2. There are two possible scenarios to explain this situation. Either the Liljeborgiidae are senticaudates and the apical robust setae on uropods 1 and 2 have been lost in the subfamily Liljeborgiinae, or alternatively the Liljeborgiidae is not a senticaudate family and apical robust setae on uropods 1 and 2 have been convergently acquired by the subfamily Idunellinae. Like liljeborgiines, Idunellines have quite lanceolate uropods 1 and 2 even though they possess small apical robust setae. In addition, when we examined the other 114 characters that were employed in our cladistic study we found that the Liljeborgiidae aligned with the suborder Amphilochoidea. We therefore found it more parsimonious to place the Liljeborgiidae in the Amphilochoidea and assume that robust setae on the apices of uropods 1 and 2 were a homoplasy in the Idunellinae.

Some of the most difficult taxa to allocate in our study (Lowry and Myers 2013) were the Eusiridae and Calliopiidae. As we stated in our paper (Lowry and Myers 2013):

*In this study, many of the clades are supported by several strong synapomorphies, but some inevitably are more weakly supported. Our aim in this work was to provide a complete classification, so we did not have the option of ignoring weakly supported clades*.

Our classification was a hypothesis and like all scientific hypotheses it is open to falsification. We do not, however, accept that the Senticaudata is falsified by the simple discovery of a few taxa outside that suborder with robust setae on the apices of uropods 1 and 2. We agree with d’Udekem d’Acoz and Verheye (2017) that the presence of robust setae on the apices of uropods 1 and 2 is homoplastic. However, their argument, that the approximately 100 families of senticaudates with robust setae on the apices of uropod 1 and 2 compose so many homoplasies for this character state, that the taxa displaying it cannot be considered monophyletic, is rejected by us. We hypothesise that the senticaudates have inherited this character state from a common ancestor and that just a very few additional taxa exhibit the character state as a homoplasy. Careful SEM studies of the apical setae of senticaudates and putative homoplastic taxa might prove informative. The Senticaudata are supported not by just one defining character state, but by our full cladistic analysis.

d’Udekem d’Acoz and Verheye (2017), claim that their earlier molecular phylogenetic analyses (Verheye et al. 2016), which focused on putative eusiroids but also included a representative sample of other amphipods did not support the validity of the suborder Senticaudata. In that study they selected 73 putative species of Eusiroidea for analysis, but they did not define on what basis these species were predetermined as eusiroid. The selection *a priori* of a eusiroid group may have affected their results. We, by contrast, did not select the Senticaudata *a priori*, it was our cladistic analysis that enabled us to recognize the clade Senticaudata. As stated in our paper (Lowry and Myers 2013): “approximately 300 characters were assembled in a DELTA database for the 212 families of world amphipods”. In that paper we presented our findings for the Senticaudata, using a subset of 41 characters. Later (Lowry and Myers 2017), we published the full analysis with a subset of 115 characters. In our full analysis we selected the Amphipoda as our ingroup. This selection was *a priori*, but we felt that it was justified by the currently universal acceptance of the Amphipoda as a monophyletic group.

In their rDNA Bayesian tree of putative Eusiroidea (Verheye et al. 2016, fig. 3) they show taxa with the senticaudate character state in the same clade as taxa without the senticaudate character state. However, contrary to their assertion that robust setae on the apices of uropods 1 and 2 show multiple homoplasies, to the extent that the character state would “appear to disappear convergently” (Verheye et al. 2016) there are actually only three families with apical robust setae on uropods 1 and 2 among the nine clades of their putative Eusiroidea. One family is represented by a single genus, *Acanthonotozoma*. The robust setae on the apices of *Acanthonotozoma* are not homologous with the senticaudate character state. As pointed out by Just (1978) in his monograph on *Acanthonotozoma*, the apices of uropods 1 and 2 have “complexly inserted spines”. They are more akin to embedded setae then they are to senticaudate apical setae. In addition *Acanthonotozoma* has character states that clearly place it in the Iphimedioidea. The second family is the Pleustidae (of which they list two examples). In this family, uropods 1 and 2 are tending towards lanceolate rather than ferrulate and uropod 3 is clearly lanceolate, a character state that does not occur in the Senticaudata. The third family, the Calliopiidae (of which they quote numerous examples) does indeed exhibit the senticaudate character state and we classified them as Senticaudata (Lowry and Myers 2013). In addition to robust setae on the apices of uropods 1 and 2 the calliopiids also have ferrulate uropods 1–2 and a distoventral robust seta on urosomite 1, both character states of senticaudates.

In such a complex and extensive analysis as ours (Lowry and Myers 2017), involving over 200 families with 1,600 genera, we would expect that some families will prove to be wrongly assigned, but this would not falsify the Senticaudata, which comprise nearly 100 families supported by many synapomorphies.

It is not clear why the “other” amphipods are considered by d’Udekem d’Acoz and Verheye (2017) to be “representative”. Representative of what? These “other” amphipods were derived from what the authors define as non-eusiroid sequences selected from the study of Englisch (2001). In what way this selection of amphipod taxa, which appear to be unrelated to their study, can test the validity of the Senticaudata is not explained. We might also add, that the relationships shown by these non-eusiroid taxa are unprecedented. For example, in their maximum likelihood tree (Verheye et al. 2016, fig. 2), *Atylus* and *Byblis* (Dexaminidae and Ampeliscidae) are sister taxa to *Bathyporeia*, (Gammaroidea); *Salentinella* (Bogidiellidae) is a sister taxon to *Haustorius* (Haustoriidae); *Stegocephalus* (Stegocephalidae) is a sister taxon to *Antatelson* (Stenothoidae) and *Syrrhoe* (Synopiidae) is a sister to *Bactrurus/Crangonyx* (Crangonyctidae). During the three centuries that scientists have turned their attention to amphipod relationships, none of these associations have ever before been suggested. Whereas our concept of the Senticaudata is not in any way revolutionary, some of the relationships suggested by Verheye et al. (2016) in their analyses are radical. Yet there is no justification provided by these authors in support of these relationships. In the analyses by Verheye et al. (2016) gene sampling was low and the two genes used were not fully congruent with each other, so criticism of any existing classification is weakly based.

Molecular studies of higher phylogeny cannot be considered as the truth. Much has still to be learned about molecular evolution. Genes can also be homoplastic. It would be statistically improbable that any given base pair substitution, occurring by random mutation in a neutral gene, could have occurred only once in evolutionary history. It is likely that homoplasy is common in the genotype as well as in the phenotype, even where neutral genes are concerned. Molecular approaches have been shown to give meaningful results at population level and may perhaps reflect real relationships at species level. However, it appears that at higher taxonomic levels the effectiveness of current molecular methods decreases, so that higher phylogenetic relationships are not reliable. This can be seen in the study by Verheye et al. (2016), where the relationships shown in their “representative sample of other amphipods” are inexplicable.
